# Psychological Well-Being in Breast Cancer: A Literature Review of the Impact of Precision Oncology and Radiogenomics

**DOI:** 10.7759/cureus.91210

**Published:** 2025-08-28

**Authors:** Aliaa M Soliman

**Affiliations:** 1 Psychological Sciences and Counseling Department, Austin Peay State University, Clarksville, USA; 2 Radiology Department, Al Adan Hospital-Ministry of Health (MOH), Kuwait, KWT; 3 Radiology Department, Shobra General Hospital, Cairo, EGY

**Keywords:** anxiety, breast cancer, depression, fear of recurrence, genetic testing, oncotype dx, precision medicine, psychological distress, psycho-oncology, radiogenomics

## Abstract

Radiogenomic testing, including tools such as Oncotype DX, MammaPrint, and Prosigna, has transformed breast cancer management through improved risk stratification and personalized therapy. However, several potential psychological burdens can result as consequences for these genomic tools. This literature review includes 25 peer-reviewed studies published between 2015 and 2025 that examined the psychological outcomes associated with radiogenomic testing in breast cancer patients. Anxiety, depression, fear of recurrence, and decision conflict were the most common themes encountered in these studies, particularly among those receiving intermediate or high genomic risk scores. Additional moderation factors such as age, health literacy, socioeconomic status, and communication quality were also linked to variations in distress levels. Thematic analysis highlights the importance of integrating psychological support, patient education, and counseling throughout the genomic testing process. This review emphasizes the need for a holistic, patient-centered approach that addresses the emotional and cognitive consequences of precision oncology and identifies key areas for future psychosocial intervention research.

## Introduction and background

In oncology, several interdisciplinary approaches are emerging, with radiogenomic testing representing one of the most advanced methods for breast cancer management. Radiogenomics integrates radiologic imaging with tumor biology and precision oncology to improve diagnosis, prognosis, and treatment planning [1,2]. Simply put, radiogenomic studies how a patient’s genes influence the appearance and behavior of their cancer on imaging (MRI, CT, or mammograms), while precision oncology uses genetic, clinical, and lifestyle information to guide individualized treatment. By linking imaging features-such as tumor shape, margin characteristics, texture, and contrast enhancement patterns-with genomic profiles, including hormone receptor status and gene expression signatures, radiogenomics enables non-invasive prediction of molecular subtypes, treatment response, and prognosis [3]. This convergence reduces reliance on invasive procedures and supports more tailored treatment strategies [4].

Historically, treatment decisions for early-stage breast cancer relied on clinicopathological factors such as tumor size, grade, nodal involvement, and receptor status. However, these markers often lacked sufficient prognostic accuracy [5]. Multigene assays such as Oncotype DX, MammaPrint, and Prosigna significantly improved recurrence prediction and assessment of chemotherapy benefit, supporting individualized, risk-adapted care that prevents overtreatment of low-risk patients while directing high-risk patients toward more aggressive therapies [6-8]. Genomic testing has, therefore, become a cornerstone of modern breast cancer care, particularly for patients with hormone receptor-positive, early-stage disease [9].

While these advances enhance clinical precision, they also carry important psychological implications. Many women undergoing genomic or radiogenomic testing experience distress related to diagnosis, prognosis, and treatment decisions, including anxiety, fear of recurrence, and uncertainty about the future [10,11]. Some patients find reassurance in low-risk scores, whereas others report heightened anxiety or decisional conflict when results suggest high-risk outcomes [12]. Communication plays a pivotal role: clear, empathic, and patient-centered discussions of genomic test results reduce anxiety and promote trust, while unclear communication can lead to confusion, regret, or disengagement from care [13,14]. Patient factors such as age, education level, socioeconomic status, and prior mental health history further shape how genomic information is understood and experienced [15].

Despite growing research on radiogenomic testing, important gaps remain. Few longitudinal studies track long-term mental health outcomes, minority and underserved populations remain underrepresented, and validated psychological assessment tools are inconsistently applied. Addressing these limitations is critical for developing equitable, patient-centered care models that integrate psychological well-being alongside clinical precision [16]. To systematically examine these issues, this review follows the PRISMA (Preferred Reporting Items for Systematic Reviews and Meta-Analyses) guidelines, ensuring transparent methods, reproducibility, and a structured evaluation of available evidence.

Purpose of this study and research question identification

The emotional and cognitive effects of radiogenomic testing on patients with breast cancer are the focus of this study. It seeks to combine the possible psychological effects, such as depression, anxiety, fear of recurrence, and conflict over treatment decisions. To investigate the relationship between radiogenomic risk scores and psychological distress, research questions were generated: (1) What are the psychological outcomes associated with radiogenomic testing in breast cancer patients? (2) How do radiogenomic-associated risk scores influence psychological outcomes? (3) How can psychosocial factors, mediators, and moderators influence psychological outcomes? (4) What are the coping strategies utilized to alleviate potential psychological outcomes? This literature review's goal is to highlight important areas for further research and to inform the best practices for a holistic patient approach that incorporates psychosocial support into precision oncology.

## Review

Materials and methods

**Figure 1:**
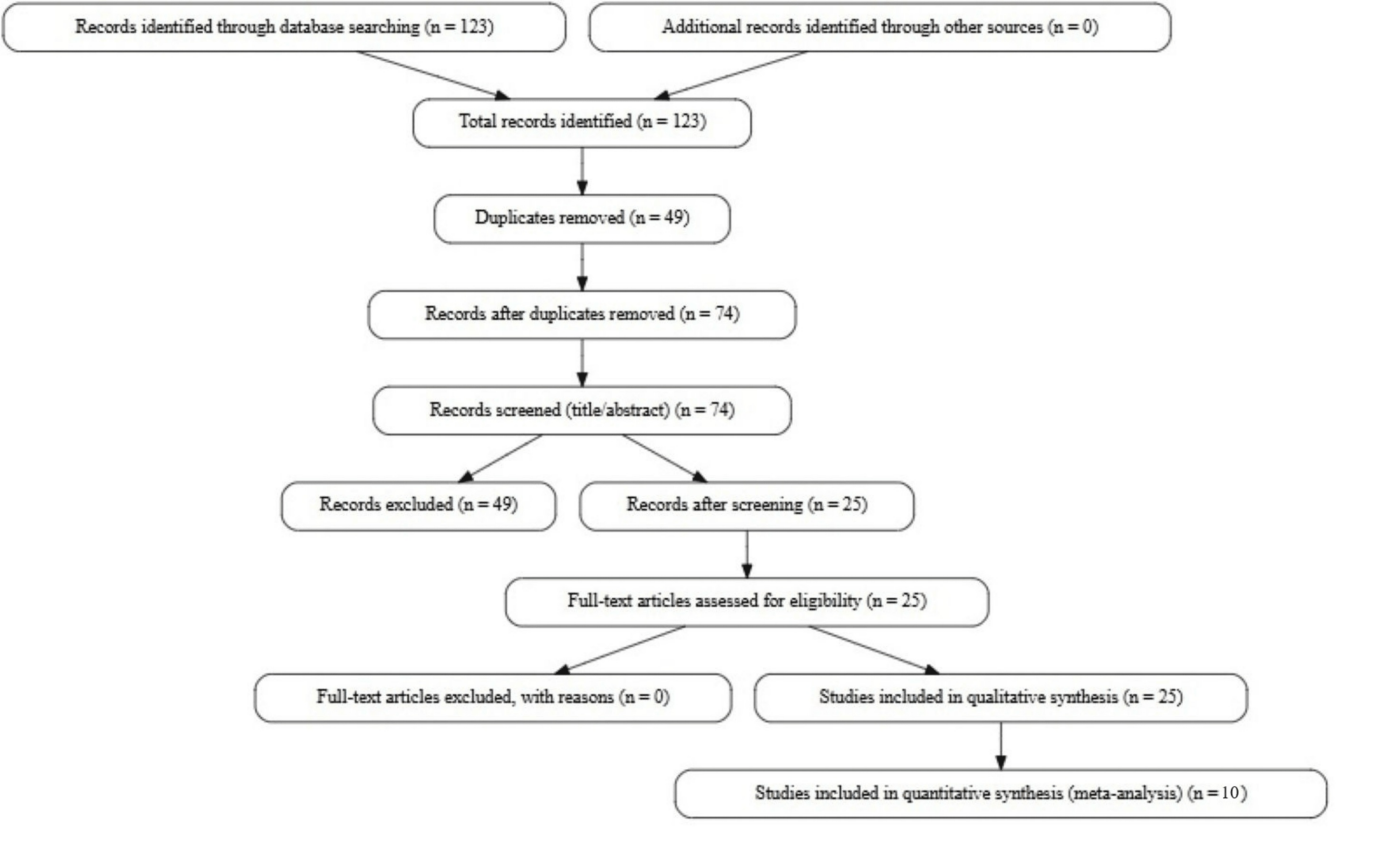
PRISMA flow diagram PRISMA: Preferred Reporting Items for Systematic Reviews and Meta-Analyses

Peer-reviewed observational studies published between 2015 and 2025 were collected and assessed using a methodical process. This review followed the PRISMA 2020 guidelines for systematic reviews. A total of 123 records were identified across six databases (PubMed, PsycINFO, CINAHL, Psycho-oncology, Web of Science, and Scopus) using a combination of keywords (“breast cancer,” “Oncotype DX,” “MammaPrint,” “radiogenomic,” “psychological distress,” “anxiety,” “depression,” and “fear of recurrence”). After removing 49 duplicates, 74 records were screened. Based on the title and abstract review, 49 records were excluded. The remaining 25 full-text articles met all inclusion criteria and were included in the review. The selection process is summarized in the PRISMA flow diagram (Figure 1).

The selected research had to meet the following inclusion criteria: it had to be about female breast cancer patients; involve radiogenomic or multigene assay testing, such as Oncotype DX, MammaPrint, or Prosigna; report psychological outcomes such as anxiety, depression, fear of recurrence, post-traumatic stress disorder (PTSD), or general distress; and be published in peer-reviewed journals in English within the specified time frame. Research that did not report psychological outcomes, was not peer-reviewed, had duplicate publications, or focused on male patients or non-breast cancer populations was disqualified.

To ensure methodological rigor and quality of the review, both qualitative and quantitative studies were included. The quality of the observational studies was evaluated using validated instruments: the Newcastle-Ottawa Scale for cohort and case-control designs and the AXIS tool for cross-sectional studies.

Results and thematic analysis

The selected 25 literature reviews were published between 2015 and 2025, with sample sizes ranging from roughly 50 to over 1,000 breast cancer patients. Women with early-stage breast cancer who underwent radiogenomic testing with Oncotype DX (n = 15), MammaPrint (n = 5), Prosigna (n = 3), and other multigene panels (n = 2) made up most of the selected populations in the reviewed research. Most studies used cross-sectional or observational cohort designs and evaluated psychological distress outcomes at different times after receiving the results of genomic tests and receiving a diagnosis, mainly depression and anxiety.

Data extraction was carried out using a standardized charting method to record study attributes, like author, publication year, country of study, participant demographics, the genomic test used, psychological evaluation instruments, and important findings (see Table 1). The results were compiled thematically to identify recurring themes, trends, and gaps in the body of knowledge about the psychological effects of radiogenomic testing in the treatment of breast cancer.

**Table 1:** Extracted data from included literatures BC: breast cancer; QoL: quality of life; PTSD: post-traumatic stress disorder

Author (year)	Radiogenomic tool	Population	Key theme	Psychological outcome	Main findings
Yanes et al. (2019) [16]	Prosigna	Postmenopausal BC patients	Decision satisfaction	Anxiety	Higher satisfaction reported with Prosigna-informed treatment plans, with reduced anxiety levels.
Tran et al. (2022) [17]	Oncotype DX	Asian BC patients	Cultural differences	Psychological distress	Asian patients reported greater distress due to limited culturally adapted counseling.
Abdel-Razeq et al. (2024) [18]	Multigene panel	BC patients with family history	Genetic uncertainty	Anxiety, distress	Family history amplified anxiety related to uncertain genomic test results.
Vajen et al. (2021) [19]	Multigene panel	Germany BC patients	Genetic uncertainty	Distress levels	Higher distress observed in individuals with pathogenic variants or variants of uncertain significance.
Oh and Son (2021) [20]	Oncotype DX	HR+/HER2- BC patients	QoL, sustainability	Fear of recurrence, QoL	Oncotype DX implementation reduced overtreatment, improved QoL, and decreased healthcare-related emissions.
Stanton and Bower (2015) [21]	Prosigna	Brazilian BC patients	QoL	Depression, anxiety	Prosigna testing linked to improved QoL and lower depression rates.
Krasne et al. (2022) [22]	Multigene panel	Younger BC patients	Fertility concerns	Anxiety, fear of recurrence	Younger patients reported high anxiety linked to fertility implications revealed by genetic tests.
Klafke et al. (2019) [23]	Oncotype DX	Early-stage BC patients	Treatment decision-making	Anxiety reduction	Patients avoided chemotherapy based on the low-risk score, leading to decreased anxiety and improved QoL.
Park et al. (2023) [24]	MRI-based RNA profiling	Korean BC patients	Predictive accuracy	Anxiety, trust in care	High predictive accuracy reduced anxiety and increased trust in clinical recommendations.
Malgaroli et al. (2022) [25]	Genetic counseling	BC survivors	Post-treatment mental health	PTSD symptoms	Genetic counseling linked to reduced PTSD symptoms among survivors receiving genomic risk information.
Omari et al. (2022) [26]	Oncotype DX	Korean BC patients	Decision-making support	Anxiety, decisional conflict	Decision aids combined with genomic results reduce anxiety and decisional conflict.
Lombardi et al. (2019) [2]	Oncotype DX	Diverse racial/ethnic groups	Test reliability across populations	Confidence in decision-making	Oncotype DX reliably predicted outcomes across racial and ethnic groups, supporting its use in diverse populations.
Maheu et al. (2019) [6]	Oncotype DX	ER+ BC patients	Long-term outcomes	Recurrence, mortality	Oncotype DX scoring accurately predicted chemotherapy benefit, with no significant difference in long-term outcomes.
Blomen et al. (2021) [7]	Oncotype DX	Diverse populations	Predictive insights	Treatment confidence	Reinforced Oncotype DX's predictive value across racial and ethnic groups, aiding in treatment decisions within high-risk breast cancer families.
Gormley et al. (2021) [3]	Machine learning model	BC patients	Predicting recurrence	Decision-making support	Machine learning models predicted Oncotype DX recurrence scores, potentially aiding in treatment decisions and reducing anxiety.
Muktar et al. (2025) [27]	Oncotype DX	Rural BC patients	Access to care	Depression	Rural patients faced higher depression rates due to limited access to genomic testing and counseling.
Kritzik et al. (2021) [8]	Genetic counseling	Newly diagnosed BC patients	Rapid genetic counseling	QoL, distress	Proactive genetic counseling improved knowledge and QoL, with no significant impact on distress.
Willis et al. (2021) [28]	MammaPrint	Early-stage BC patients	Treatment adherence	Depression	Patients with low MammaPrint risk showed higher treatment adherence and lower depression scores.
Brédart et al. (2022) [29]	Genetic counseling	Hispanic BC patients	Family involvement	Social support, anxiety	Family involvement improved social support and reduced anxiety in genomic testing decisions.
Lamontagne (2023) [13]	Oncotype DX	Latina BC patients	Communication quality	Decisional conflict	Clear communication reduced decisional conflict and improved psychological outcomes.
Al-Alawi et al. (2022) [30]	Oncotype DX	Elderly BC patients	Treatment toxicity concerns	Anxiety, depression	Elderly patients reported anxiety related to fears of treatment toxicity guided by genomic risk.
Macias-Konstantopoulos et al. (2023) [31]	Oncotype DX	African American BC patients	Health disparities	Depression, distress	Disparities in test access linked to increased psychological distress in African American women.
İzci et al. (2016) [32]	Not specified	BC patients	Mental health impact	Anxiety, depression	Breast cancer diagnosis significantly impacts mental health; coping strategies and support systems are crucial.
Sullivan et al. (2025) [33]	Oncotype DX	US BC patients	Temporal trends	Chemotherapy usage	Increased Oncotype DX use associated with decreased chemotherapy usage and improved survival rates.
American Family Physician (2020) [34]	Genetic counseling	BC patients with BRCA mutations	Psychological adaptation	Anxiety, adjustment disorder	Counseling helped patients adapt psychologically to high genetic risk status.

Theme I: Potential Psychological Challenges Associated With Radiogenomic Testing

In the reviewed literature, anxiety, depression, and fear of recurrence are the most measured psychological outcomes related to radiogenomic testing. Reliable measures of these symptoms, such as the Hospital Anxiety and Depression Scale (HADS) and the Patient Health Questionnaire (PHQ-9), have been used in numerous studies to measure distress levels [16,17]. Yanes et al. and Tran et al. both emphasized that the breast cancer Prosigna testing genotype reported higher satisfaction with Prosigna-informed management plans, with reduced anxiety levels. On the other hand, Abdel-Razeq et al. studied the psychological impact of a multigene panel among breast cancer patients with a strong family history and concluded that genetic uncertainty significantly increases anxiety and emotional distress, particularly in individuals with a strong hereditary tendency [18]. Therefore, positive family history seems to amplify psychological responses to inconclusive test results, underlining the need for targeted genetic counseling and emotional support in this subgroup [18,19]. Furthermore, other research has been done on the fear of cancer recurrence (FCR), which is a major concern for patients, particularly those with higher genomic risk scores [6,20]. Despite being researched less, some longitudinal studies highlight the long-term psychological effects on this population by displaying symptoms like PTSD [21].

Studies consistently showed that patients with high or intermediate genomic risk scores experienced noticeably higher levels of psychological distress than patients with low-risk scores. At least 18 studies found that patients with intermediate- or high-risk genomic profiles had higher levels of anxiety, making it the most measured psychological outcome [8,22,23]. For example, three months after testing, patients with high Oncotype DX scores were 2.5 times more likely to have clinically significant anxiety symptoms, according to a large cohort study (N = 800) [16].

Twelve studies reported depressive symptoms, and while the extent and consistency of these effects varied more than those for anxiety, they generally indicated a trend toward higher depression scores in higher-risk patients [6,24]. Numerous studies have reported symptoms of PTSD, such as intrusive thoughts and avoidance behaviors, with higher-risk patients exhibiting more severe symptoms [25,26].

According to 10 studies, FCR was a major psychological concern. The findings consistently demonstrated a strong positive relationship between fear of recurrence and genomic risk categories, with intermediate- and high-risk patients expressing concerns that persisted for longer than a year after the end of treatment [17,20].

Theme II: Relationship Between Psychological Distress and Various Radiogenomic Risk Scores

In general, patients with higher genomic risk scores report higher levels of anxiety and depressive symptoms, according to the association between psychological distress and radiogenomic risk scores, specifically Oncotype DX and MammaPrint [27]. For instance, people with intermediate to high Oncotype DX scores showed noticeably more distress than people with low-risk results, according to Malgaroli et al. and Willis et al [25,28]. On the other hand, Brédart et al. pointed out that the uncertainty and stress of decision-making, regardless of the risk level, might cause distress when receiving any genomic risk result [29]. Early evidence suggests a similar pattern, where higher-risk categories correlate with increased distress, even though fewer studies have looked at MammaPrint scores [24]. Nevertheless, the growing adoption of Oncotype DX among breast cancer patients in the United States appears to induce a significant reduction in chemotherapy usage, resulting in improved survival outcomes, which indicate the clinical utility of the Oncotype DX test in promoting personalized treatment while minimizing overtreatment and improving the general well-being [33].

The timing of psychological assessment also appeared to be crucial in the identification of the induced psychological effect. Studies differed widely when psychological evaluations were conducted, ranging from right after disclosure of radiogenomic results to years after survivorship. Peak distress, particularly anxiety and fear of recurrence, was usually observed in early post-test evaluations (within three months) [20]. A significant portion of intermediate- and high-risk patients continued to have elevated anxiety and fear after 12 months, even though distress generally decreased over time [3,28]. However, the research of Gormley et al. highlighted a machine learning model that provides patients and clinicians with early data-driven insights to recurrence risk, which has proven potential reduction of the initial anxiety surrounding uncertainty in prognosis, indicating alleviated FCR with built confidence in the tailored treatment approach [3].

Theme III: Psychosocial Mediators and Moderators

Psychosocial factors have been found in several studies to mediate or moderate the relationship between psychological distress and genomic risk. Adequate counseling and clear communication of results seem to lessen the potential negative psychological effects [7], indicating that supportive care is crucial in the management of genomically informed breast cancer.

The strongest protective factor was found to be social support, especially from peers and family, which mitigated distress irrespective of the level of genomic risk [26,28]. Psychological outcomes were also strongly impacted by health literacy and patients' understanding of test results; greater understanding was associated with lower anxiety and more confidence in treatment [8,30]. Furthermore, Lamontagne conducted research among Latino breast cancer patients and concluded that effective communication with patients not only supported informed treatment choices but also improved psychological outcomes, which helped the patients feel more confident and emotionally secure throughout the decision-making process [13]. This research highlighted the significance of clear, culturally sensitive communication in reducing decisional conflicts for breast cancer management [13].

Patients' psychological reactions to radiogenomic testing have been found to be significantly influenced by additional psychosocial factors, such as cultural background, family involvement, and communication quality [8,29]. These components draw attention to differences in psychological outcomes and point to areas that could benefit from specialized interventions to enhance mental health care. To help patients manage their anxiety, depression, PTSD symptoms, and fear of recurrence, the current body of evidence supports the integration of strong psychological support throughout the genomic testing process [2]. Also, Macias-Konstantopoulos et al. concluded in their research that this will ultimately improve treatment adherence and quality of life [31].

Theme IV: Coping Strategies and Interventions

The reviewed literatures showed a recurring theme about the most popular methods to alleviate anxiety and improve coping mechanisms among breast cancer patients. Yanes et al. and Muktar et al. examined cognitive-behavioral therapy (CBT) and mindfulness-based stress reduction (MBSR), particularly for patients with high-risk genomic results [16,27]. Additionally, Al-Alawi et al. discussed how arming patients with information and elucidating treatment options, patient education, and counseling interventions have an effective outcome in lowering emotional distress and decisional conflict [30]. Peer counseling and support groups have also demonstrated positive impacts on social connectedness and emotional well-being [29]. Also, İzci et al. emphasized that effective coping strategies like mindfulness techniques and exercises besides a strong support system are essential for maintaining emotional well-being throughout the cancer management journey [32]. Another method for alleviating induced psychological distress is genetic counseling [18,34]. An article published in American Family Physician (2020) reported that genetic counseling played a significant role in supporting breast cancer patients with BRCA mutations, facilitating psychological adaptation to their elevated genetic risk. The intervention was associated with reduced anxiety levels and improved emotional adjustment, helping patients better cope with the implications of their BRCA-positive status and reducing the risk of developing adjustment disorders [34].

Discussion

A distinct pattern emerges from the review's combined data: patients with breast cancer who have intermediate or high radiogenomic risk scores typically endure noticeably more psychological distress than those who have low-risk results. The most prevalent and immediate emotional reaction after learning one's genetic risk status seems to be anxiety. The uncertainty surrounding a higher chance of recurrence and the consequences for more intensive or drawn-out treatment plans are probably the root causes of this anxiety. According to studies like Yanes et al. and Klafke et al., the psychological effects can last throughout the crucial decision-making phase that follows diagnosis and are not just a temporary reaction [16,23].

Numerous studies have documented a strong fear of recurrence, which emphasizes how genomic testing, although medically advantageous, also imposes a psychological burden that may impair patients' everyday functioning and quality of life. Relationship stress and general well-being may result from this fear, which frequently shows up as hypervigilance to physical symptoms, sleep issues, and elevated health anxiety [17,20]. These results lend credence to the idea that, rather than being an optional supplement, psychological support ought to be regarded as an essential part of genomically informed care.

It is interesting to note that PTSD and depression symptoms were not as consistently linked to genomic risk scores. This variability could be explained by variations in measurement instruments, sample characteristics, and assessment timing among studies. For instance, a subset of patients who already have vulnerabilities or who encounter other stressors in their lives that are unrelated to their cancer prognosis may develop depressive symptoms. People who have had traumatic medical experiences in the past or who are more sensitive to stress related to their health may develop symptoms of PTSD [25]. These subtleties show how complicated the psychological landscape is and imply that when treating mental health needs, clinicians need to take an individualized approach.

Commonly Measured Psychological Outcomes

The two psychological outcomes that are most frequently measured in breast cancer patients undergoing radiogenomic testing, according to the reviewed literature, are anxiety and depression, with additional focus on the fear of recurrence. To accurately measure these symptoms, several studies used validated tools like the PHQ-9 and the HADS [16,17]. A major concern for patients, particularly those with higher genomic risk scores, is the FCR, which is also widely discussed in the literature [6,20]. Despite less research, some longitudinal studies have reported symptoms that are consistent with PTSD, highlighting the possibility of long-term psychological consequences in this population [21]. Ultimately, the reviewed literatures emphasize the correlation between psychological distress and radiogenomic testing data related to breast cancer prognosis, specifically anxiety and fear of recurrence.

Psychological Distress and Radiogenomic Risk Score Correlation

In general, patients with intermediate or high genomic risk scores report more anxiety and depressive symptoms than those with low-risk scores, according to the correlation between psychological distress and radiogenomic risk scores, mainly Oncotype DX and MammaPrint. For instance, Malgaroli et al. and Willis et al. concluded that patients with intermediate to high Oncotype DX scores showed noticeably greater distress [25,28]. On the other hand, Brédart et al. stress that the inherent uncertainty and stress associated with genomic test results and treatment decision-making can cause distress regardless of the particular risk category [29]. Emerging data point to a similar pattern of elevated distress among higher-risk patients, even though fewer studies have looked at MammaPrint risk scores [24]. Crucially, psychosocial elements like clear test results, communication, and sufficient counseling seem to mitigate these negative psychological impacts, highlighting the significance of supportive care in genomically informed breast cancer treatment [7].

Time and Longitudinal Factors

A key factor in clinical intervention is the timing of psychological distress after radiogenomic testing. Most studies found that anxiety and recurrence fear peaked shortly after test results were received, which corresponded with increased uncertainty and treatment planning [3]. Targeted psychosocial support may be able to stop the development of chronic distress or psychiatric comorbidity during this vulnerable window. For some patients, fear of recurrence often lasts for a long time, even though anxiety usually decreases over months as they get used to their prognosis and treatment results. Chronic fear can affect physical health, follow-up care compliance, and quality of life [20]. This research backs up the necessity of regular evaluations of psychological health in survivorship care, along with prompt referrals to mental health specialists for individuals exhibiting persistent distress [20].

Clinical Implications and Psychosocial Moderators

Numerous studies have highlighted how important psychosocial moderators are in influencing psychological outcomes, including coping strategies, social support, and health literacy. According to research by Balitsky et al. and Willis et al., patients who had strong social networks-such as friends, family, and peer support groups-reported feeling less distressed [1,28]. Social support can reduce anxiety and promote resilience by offering practical help, emotional reassurance, and a barrier against feelings of loneliness [35].

Another crucial element that surfaced was health literacy. Patients are better able to make decisions and experience less helplessness and uncertainty when they are fully informed about their genomic results and the implications for treatment options [8]. Educational interventions can enhance comprehension and reduce distress by personalizing risk communication and elucidating the meaning of tests [30].

Coping mechanisms also have a big impact on results. Avoidant coping, which is characterized by denial or disengagement, predicted worse emotional adjustment, according to Omari et al., while active coping strategies, such as information-seeking, problem-solving, and emotional expression, were associated with lower psychological distress [26]. These results lend credence to the idea that psycho-oncology services should include coping skills training to help patients cope with the psychological effects of their diagnosis and genetic risk.

*Coping Strategies and Interventions*****

Although there is still a dearth of intervention research in this area, recent studies show encouraging methods for helping patients understand the results of radiogenomic tests [2]. The most widely used strategies, psychoeducation, MBSR, and CBT, have shown promise in lowering anxiety and enhancing coping mechanisms, especially for patients with high-risk genomic results [16,27]. Furthermore, by increasing knowledge and making treatment options clear, patient education and counseling interventions have been demonstrated to lessen emotional distress and decisional conflict [30]. Peer counseling and support groups also have a positive impact on social connectedness and emotional well-being [19]. To develop standardized psychosocial interventions in the framework of radiogenomic-informed care, however, more thorough and extensive clinical trials are required [18].

Limitations and future directions

Several limitations inherent in the existing literature must be acknowledged when interpreting the findings of this review. First, the heterogeneity in study designs, sample characteristics, and psychological outcome measures complicates synthesis and precludes the possibility of robust meta-analytic aggregation. Many studies predominantly relied on self-report questionnaires, which are vulnerable to response biases such as social desirability or recall inaccuracies. Additionally, most of the research has focused on early-stage female breast cancer patients, limiting the generalizability of findings to patients with advanced disease stages, male breast cancer patients, or individuals with other cancer types where genomic testing is increasingly utilized. Future research should prioritize longitudinal study designs that track psychological distress trajectories from diagnosis through survivorship to better elucidate causal pathways and identify critical windows for intervention. Expanding demographic and clinical diversity within study samples-including male patients, advanced-stage cancer, and diverse ethnic and socioeconomic backgrounds-will enhance the external validity of findings. Furthermore, well-designed randomized controlled trials evaluating psychosocial interventions tailored specifically to genomic risk communication are urgently needed to provide evidence-based guidelines for integrating mental health support into genomic-informed cancer care.

## Conclusions

Radiogenomic testing tools such as Oncotype DX, MammaPrint, and Prosigna have revolutionized personalized breast cancer care by enabling more precise, tailored treatment decisions that improve clinical outcomes. However, this review highlights the significant psychological impact these genomic risk scores impose on patients, particularly through heightened anxiety and FCR. The emotional burden associated with receiving intermediate- or high-risk genomic results underscores the critical need to integrate psychosocial care into standard oncology practice. Routine mental health screening, comprehensive patient education, and supportive psychosocial interventions should be embedded alongside genomic testing protocols. Clear, compassionate communication regarding risk results, combined with strategies to foster adaptive coping, can effectively mitigate distress and empower patients during their treatment journey. Given the persistence of fear of recurrence and the variability in depressive and trauma-related symptoms, psychosocial support must extend beyond the immediate diagnostic and treatment phases into long-term survivorship care. Multidisciplinary collaboration involving oncologists, mental health professionals, genetic counselors, and patient navigators is essential to address the multifaceted needs of breast cancer patients in the genomic era. Ultimately, genomic information is not solely clinical data but deep personal knowledge that shapes patients’ lived experiences. Bridging precision oncology with compassionate psychosocial care enables healthcare providers to support patients more holistically, improving both medical outcomes and quality of life. This review emphasizes that anxiety, depression, and fear of recurrence are the most prominent psychological outcomes linked to radiogenomic risk scores, with higher scores generally correlating with greater distress. Effective communication and psychosocial support emerge as critical moderators of these outcomes. Although interventions such as CBT, mindfulness, and enhanced patient education show promise, the current evidence base remains insufficient to establish definitive clinical guidelines. Future research should focus on large-scale psychosocial intervention trials and explore less frequently studied outcomes like post-traumatic stress symptoms to inform comprehensive care models. Integrating psychosocial support into radiogenomic testing pathways is vital to optimize both psychological well-being and oncologic outcomes for breast cancer patients.
